# Radical Treatment of Diseases of Nose and Throat

**Published:** 1895-10

**Authors:** F. M. Reeves

**Affiliations:** Houston Infirmary; Houston, Texas


### RADICAL TREATMENT OF DISEASES OF NOSE AND THROAT.

HOUSTON, TEXAS, August 24, 1895.

*Atlanta Medical and Surgical Journal:*

Conscientious physicians are always ready to say, when asked as to their success in treating these diseases, that those of the nose, especially, are very intractable. They consequently waste but little time and attention on them, and expect to only alleviate them as a rule. I have been giving the subject the very closest attention for the past few years, and notwithstanding my poor success, until the last few months clung to it most tenaciously.

During my last visit to New York in quest of something new, my attention was called to a preparation that I have since used as a basis, and with the most gratifying results. Have used it in atrophic and hypertrophic rhinitis and "post nasal catarrh" and pharyngeal affections. I usually prescribe it as follows:

℞ Trikresoliodin, ℥ i.
Sig. Spray nose and throat morning and evening.

Though in pharyngeal diseases have gotten better results by touching up surfaces thoroughly. It is very hygroscopic, and cannot be used in metallic instruments. It is heavy with chlorides evidently. I use Aloe's ointment atomizer.

It secures a splendid reaction, and is a good antiseptic. No undue irritation is produced. In fact, I use it in absolute strength as prepared. Treated a case of atrophic rhinitis—young lady ; the mucous surface seemingly sclerosed and ulcerated. She was discharged after having only three weeks' treatment. The sense of smell had been destroyed entirely ; she recovered that, too.

The regular physician should give these diseases good attention; why send them to a specialist ? You can treat them as successfully and get good fees for it. Give the preparation a trial, and report same through your journal. Everything with merit in it should be given to the profession. F. M. REEVES, M.D.

*Houston Infirmary.*

---

### CLINICAL VALUE OF RENAL CASTS.

Many clinicians can recognize casts under the microscope but fail to interpret their significance. Dr. A. E. Austin, in the *International Medical Magazine,* separates them into definite groups, and draws inferences accordingly. The hyaline and blood casts merely indicate irritation and hyperemia. Closely associated with hyaline casts is amyloid change of the kidney, due usually to suppuration, and with a good prognosis if the source of suppuration be removed. Epithelial, granular, and fibrinous casts indicate acute inflammation of the renal tubules, the epithelial cast pointing to the mild stage ; that is, one of mere desquamation with urine slightly under normal in amount, specific gravity 1030, high color, albumin a trace, numerous hyaline casts, free blood, and renal epithelium. This may go on to brown or pale granular casts with diminution of the solids of the urine. The fatty and waxy casts, when numerous and persistent, indicate long-continued chronic inflammation of the kidney, with bad prognosis. Blood and epithelial cells are absent. In this case the urine is diminished in amount with increased specific gravity. The pale, granular and small hyaline casts point to fibroid kidney. Aside from the microscopical appearance, the general condition of the patient, age, sex, and previous history must be taken into consideration. It should be remembered that the diagnosis of cases alone may mean little, but the kind of casts in abundance is the important point.—*Maryland Med. Jour.*

